# Application of Machine Learning to Diagnostics of Schizophrenia Patients Based on Event-Related Potentials

**DOI:** 10.3390/diagnostics13030509

**Published:** 2023-01-30

**Authors:** Nadezhda Shanarova, Marina Pronina, Mikhail Lipkovich, Valery Ponomarev, Andreas Müller, Juri Kropotov

**Affiliations:** 1 Theoretical Cybernetics Department, Saint Petersburg State University, 198504 St. Petersburg, Russia; 2 N.P. Bechtereva Institute of the Human Brain of the Russian Academy of Sciences, 197376 St. Petersburg, Russia; 3 Institute of Problems in Mechanical Engineering, 199178 St. Petersburg, Russia; 4 Brain and Trauma Foundation, CH-7000 Chur, Switzerland

**Keywords:** electroencephalogram, event-related potential, schizophrenia, machine learning models, support vector machine

## Abstract

Schizophrenia is a major psychiatric disorder that significantly reduces the quality of life. Early treatment is extremely important in order to mitigate the long-term negative effects. In this paper, a machine learning based diagnostics of schizophrenia was designed. Classification models were applied to the event-related potentials (ERPs) of patients and healthy subjects performing the visual cued Go/NoGo task. The sample consisted of 200 adult individuals ranging in age from 18 to 50 years. In order to apply the machine learning models, various features were extracted from the ERPs. The process of feature extraction was parametrized through a special procedure and the parameters of this procedure were selected through a grid-search technique along with the model hyperparameters. Feature extraction was followed by sequential feature selection transformation in order to prevent overfitting and reduce the computational complexity. Various models were trained on the resulting feature set. The best model was support vector machines with a sensitivity and specificity of 91% and 90.8%, respectively.

## 1. Introduction

Schizophrenia is a complex, heterogeneous behavioral and cognitive syndrome that seems to originate from the disruption of brain development caused by genetic or environmental factors, or both [1]. It is extremely important to treat schizophrenia as soon as possible after onset. With a delay in effective treatment, patients may be at increased risk for brain volume loss with adverse implications for long-term treatment outcomes [2]. Schizophrenia is diagnosed mainly using standard psychiatric questionnaires and clinical interviews. Due to these reasons, various tools that could help psychiatrists to diagnose schizophrenia are of huge importance.

It is well-known that the decline in cognitive function is the core symptom of schizophrenia [3]. Most patients with schizophrenia will report executive attention deficits and a significant decrease in goal-directed behavior [4,5], including poor inhibitory control [6] and altered error- and conflict-monitoring ability [7]. Cognitive control impairments in schizophrenia have been associated with disturbances in the fronto-striatal brain network including the dorso-lateral, ventro-medial prefrontal cortex, and anterior cingulate cortex (ACC). Abnormal dopamine transmission may contribute to this dysfunction, as L-DOPA administration and dopamine depletion are associated with changes in the fronto-striatal connectivity [8]. The main cognitive manifestations that suggest prefrontal dysfunction are deficits in response initiation and suppression, focused attention, rule deduction, and problem solving as well as difficulties in planning, information generation, maintaining a response pattern, and changing a response pattern to another. Patients also exhibit disorders of sustained attention, task coordination, and divided attention, related memory disorders as well as deficit in social cognition expressed as difficulties in understanding the mental states of others and attributing intentions [4].

One of the most important executive functions that is affected in schizophrenia is response inhibition, which underlies behavioral flexibility by allowing highly automated, yet contextually inappropriate, actions to be stopped [9]. The processes associated with inhibition are considered to be critical for top–down cognitive control and its translation into real everyday behavior including self-regulation and the regulation of emotions. In addition, inhibition-related features are essential for efficient working memory, restricting access to and deleting information that is no longer needed [10].

Poor inhibitory control in schizophrenia has typically been associated with reduced activation in the lateral prefrontal cortices during response inhibition in Go/NoGo and stop-signal tasks [6], reduced sensorimotor and supplementary motor cortex (SMA) activity during movements [11], and altered pre-SMA/SMA activity during proactive motor inhibition tasks [6].

Since cognitive functioning reflected in the parameters of spontaneous and event-related EEG activity, one of the possibilities of improving the reliability of diagnosis is to apply machine learning to the EEG data in order to separate individuals with schizophrenia from a control group. In [12], it was observed that the EEG of patients with schizophrenia had lower complexity values compared to healthy people, especially in the left frontal (F3) and parietal (P3) regions, which could be used for classification. EEG complexity analysis from [13] led to a similar conclusion. In [14], patients performed a sensory task and features extracted from the event-related potentials (ERP) were used as the input to the machine learning models.

In the present work, we applied machine learning models to the event-related potentials of subjects performing a modification of the visual cued Go/NoGo task [15,16]. Tests of the Go/NoGo paradigm are widely used in research on cognitive functions [17]. This type of task requires responding (for example, by pressing a button) to certain stimuli and refraining from pressing others. Previous studies of schizophrenia patients in this and similar task paradigms have shown a decrease in the amplitude of P300 and contingent negative variation (CNV) ERP waves, associated with cognitive control processes [18,19,20,21]. In the present study, we selected 18 ERP signals of 200 ms on average in the four test conditions for the analysis. Intervals and topography of the selected signals corresponded to the P300 and CNV waves.

A set of simple features was extracted from these signals such as average, maximum, and minimum values. Features were extracted using sliding windows analogously to [22] and optimal parameters of these windows were found using grid-search procedure along with the model hyperparameters. In order to prevent models from overfitting by reducing the model complexity, sequential features selection was applied to the ERP features. These features were combined with additionally recorded behavior data, which led to an improvement in performance. Various models were evaluated on the extracted features.

A similar approach to the classification of ADHD was implemented in [22,23].

## 2. Materials and Methods

### 2.1. Subjects

Data from 132 healthy subjects (53 males, mean age 31.8 ± 8.26; range 18–50) were included in the study. Healthy subjects were recruited during the HBImed Database Collection Project in Chur, Switzerland, at the Saint Petersburg State University, and the N.P. Bechtereva Institute of the Human Brain RAS. None of the subjects had a neurological history including head injuries with loss of consciousness or any systemic medical illness diagnoses. None of the subjects were taking medication at the time of the EEG recording. Subjects were tested within a single session lasting approximately two and a half hours. During this session, a series of questionnaires such as the Brief Symptom Inventory [24], Health History questionnaire, and Current Symptoms Scales [25] were filled out. Furthermore, the control subjects had to score lower than the level of clinical significance on the symptom checklists.

The schizophrenic group consisted of 68 patients (47 males, mean age 30.6 ± 7.17; range 18–50). Patients were diagnosed on the basis of a clinical interview by senior staff psychiatrists at the psychiatric department of the Clinic of the N.P. Bechtereva Institute of the Human Brain RAS and met the ICD-10 criteria for the diagnosis of schizophrenia at least 6 months prior to the study. All patients presented with moderate to severe degree of social and cognitive impairment at the time of EEG recording. The staff psychiatrists also assessed the participants’ positive and negative symptom severity by using the positive and negative symptoms scale (PANSS) [26]. The mean value of the negative symptoms scale was 20.1 ± 4.4 (mean ± SD) and the positive symptoms scale was 26.1 ± 4.6. On the general psychopathology scale, the patients had a score of 47.9 ± 5.9. Eighteen patients received atypical antipsychotic medication (risperidone, aripiprazole, clozapine, quetiapine, amisulpride), 19 patients received typical antipsychotic medication (Haloperidol), and 31 patients received no neuroleptic medication. 

Groups of participants did not differ significantly in age, gender, handedness, and years of education.

The study was approved by the local ethics committee and written informed consent was obtained from all participants after an explanation of the procedure.

### 2.2. EEG Recording and Processing

A 19-channel EEG was recorded in the frequency band of 0.53–30 Hz and sampling at a frequency of 250 Hz. The electrodes were placed according to the International 10–20 system in the Fp1, Fp2, Fz, F3, F4, F7, F8, C3, Cz, C4, T3, T4, P3, Pz, P4, T5, T6, O1, O2 places. The electrode resistance did not exceed 5 kΩ. The EEG was recorded relative to the physically connected ears. The recordings of healthy subjects and patients used the same equipment and software under the same conditions. The equipment included the Mitsar-201 electroencephalographic system (KE 0537), controlled by a PC, manufactured by Mitsar LLC (http://www.mitsar-eeg.com, accessed on 1 January 2022), electrode cap ELECTROCAP with 19 tin electrodes (http://www.electro-cap.com/caps.htm, accessed on 1 January 2022), ECI ELECTRO-Gel for electrode contact with the scalp, and skin prep gel NuPrep for preparing the skin of ear lobes (https://www.weaverandcompany.com/, accessed on 1 January 2022). The software included the WinEEG program (https://mitsar-eeg.com/eeg-system-solutions/wineeg-research-software/, accessed on 1 January 2022) for EEG collection and analysis and Psytask software for the stimulus presentation.

Blink artifacts were corrected by independent component analysis (ICA) applied to the raw EEG fragments (i.e., by the zeroing of independent component activation curves associated with individual eye blinks as described in [27]). Furthermore, to eliminate other types of artifacts (muscle tension, lateral eye movements, slow drifts, etc.), epochs with an EEG amplitude above the threshold were automatically excluded from further analysis. The thresholds were set as follows: (1) 100 μV for all EEG frequencies; (2) 50 µV for slow EEG waves in the range of 0.53–1 Hz; and (3) 35 µV for fast frequency waves for EEG in the range of 20–35 Hz. These threshold values were selected empirically by multiple processing with different parameters and subsequent visual analysis of the results [28].

The ERPs were calculated for each channel, each type of trial, and each subject separately.

### 2.3. Experimental Setup

A modification of the visual cued Go/NoGo paradigm described in [15,16] was used. Three categories of visual stimuli were selected: (a) 20 different images of animals, referred to as “A”; (b) 20 different images of plants, referred to as “P”; (c) 20 different images of people of different professions, and this was presented together with an artificial “novel” sound, referred to as “H.” All visual stimuli were selected to have similar size and luminosity. The randomly varying novel sounds consisted of five 20-ms fragments filled with tones of different frequencies (500, 1000, 1500, 2000, and 2500 Hz). Stimulus intensity was about 70 dB SPL, measured at the patient’s head.

The trials consisted of presentations of two stimuli with an exposition of 100 ms, interstimulus interval of 1000 ms, and inter-trial intervals of 3000 ms. Four categories of trials were used: A-A, A-P, P-P, and P-H. The trials were grouped into four blocks with 100 trials each. In each block, a unique set of five A, five P, and five H stimuli was selected. Each block consisted of a pseudo-random presentation of 100 pairs of stimuli with equal probability for each stimulus category and for each trial category.

Participants practiced the task before the recording started. Subjects rested for a few minutes after each 200 trials. Subjects sat upright in a comfortable chair looking at a computer screen. The instruction was to press a button with the right hand to all A-A pairs as fast as possible and to withhold from button pressing the other pairs.

### 2.4. Event-Related Potentials

**Figure 1:**
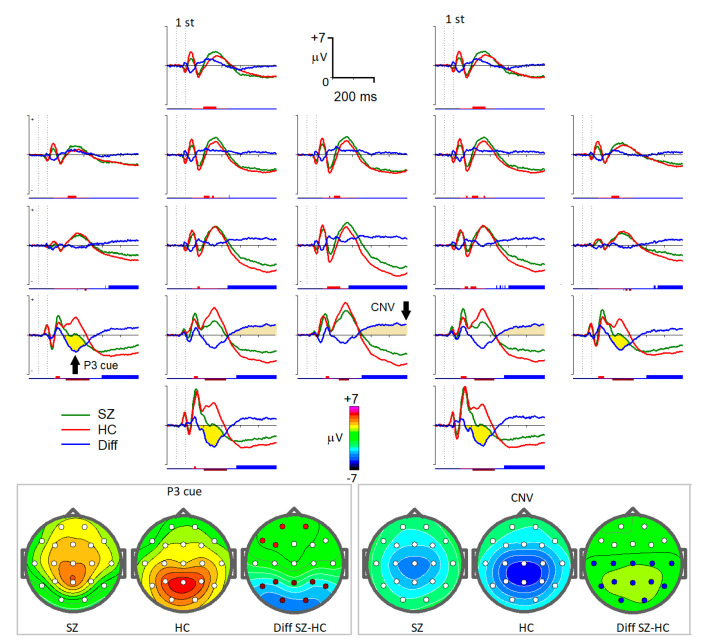
Grand average 19-channel ERPs for the two groups for the cue condition when the first stimulus in a trial was an image of the animal and the subjects had to prepare for the following stimulus. Green lines—patients with schizophrenia (SZ). Red lines—healthy controls (HC). Blue lines—difference waves SZ-HC. Marks below ERPs on each channel—statistically significant (*p* < 0.05) differences between the clusters (indicated by different colors).

**Figure 2:**
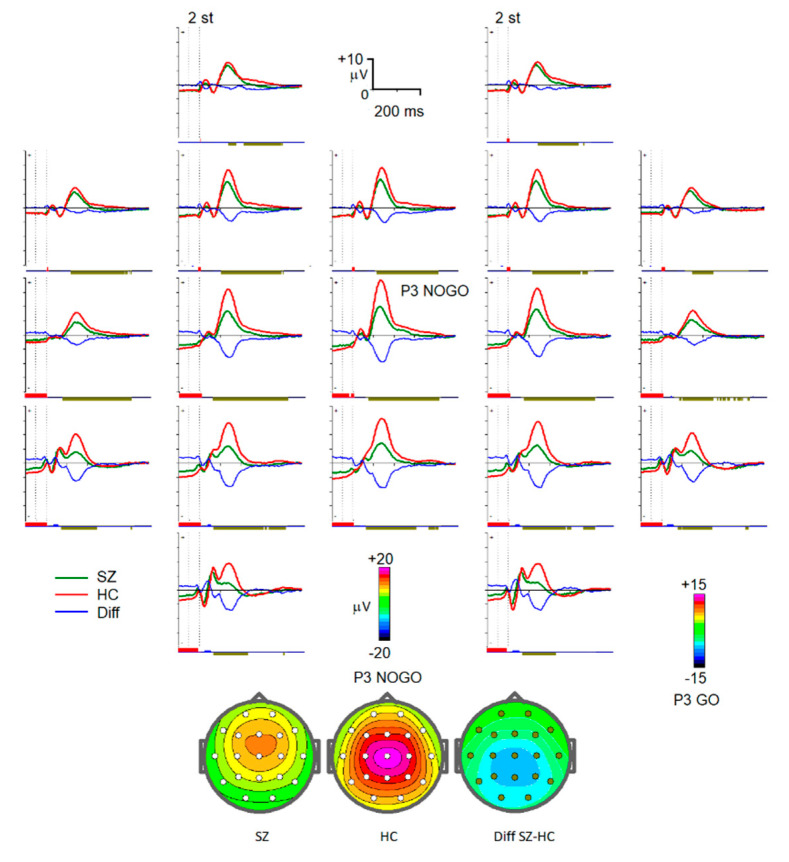
Grand average 19-channel ERPs for the two groups for the NoGo condition. Abbreviations are the same as in Figure 1.

**Figure 3:**
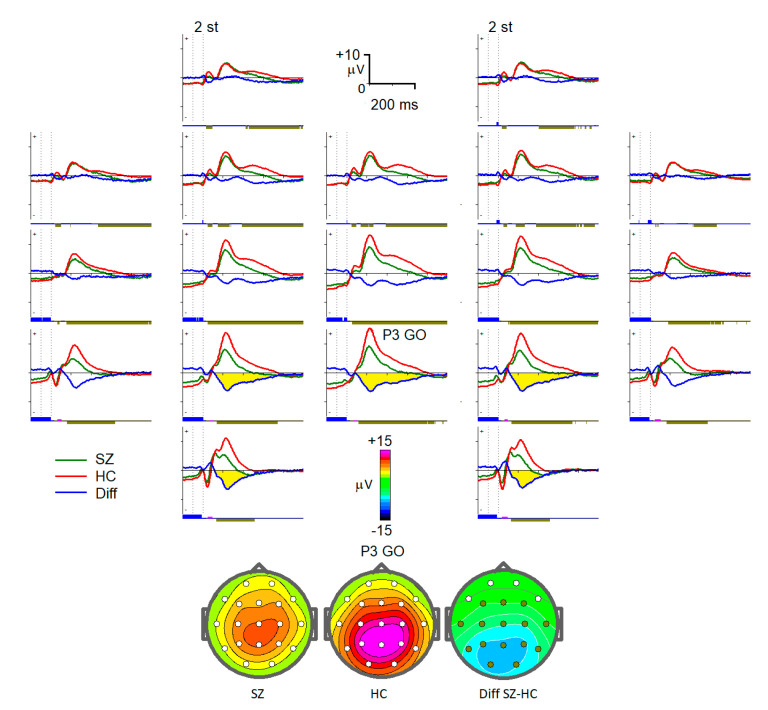
Grand average 19-channel ERPs for the two groups for the GO condition. Abbreviations are the same as in Figure 1.

Event-related potentials were selected for the groups of healthy individuals and individuals with diagnosed schizophrenia in the following conditions:

- ‘Plus1’: Condition with an image of an animal as the first stimulus in the trial and arbitrary second stimulus. For channels T5, O1, O2, and T6, the interval from 320 ms to 520 ms after the first stimulus was taken. This interval and localization corresponded to the P3Cue wave of the ERPs (Figure 1) [29,30].

- ‘Plus2’: Condition with an image of an animal as the first stimulus in the trial and arbitrary second stimulus. For channels P3, Pz, and P4, the interval was from 900 ms to 1080 ms. The selected interval corresponded to the contingent negative variation (CNV) wave of the ERPs observed just before the expected stimulus presentation (Figure 1) [31].
- ‘NoGo’: Condition with an image of an animal as the first stimulus and an image of a plant as the second one. Selected channels were C3, Cz, C4, P3, Pz, and P4 with an interval from 300 ms to 500 ms after the second stimuli (Figure 2).

- ‘Go’: Condition with an image of an animal as both stimuli. Channels of interest were C3, Cz, C4, P3, Pz, and P4 with an interval from 250 ms to 450 ms after the second stimuli. Selected intervals for NoGo and Go conditions corresponded to the P300 wave for NoGo stimuli (P300 NOGO) and P300 wave for Go stimuli (P3b) observed within 300–500 ms over the frontal and parietal cortex correspondingly (Figure 2 and Figure 3) [32,33,34].

- ‘P-H’: Condition with an image of a plant as the first stimulus and an image of a human as the second stimulus. Selected channels were C3, Cz, and C4 with an interval from 160 ms to 220 ms after the second stimuli. The interval and localization corresponded to P3a wave elicited by infrequent unpredictable stimuli [32].

### 2.5. Behavioral Data

Aside from the collection of EEG data during the experiment, the subject’s responses were also recorded using a separate channel. The response was considered correct if it was made within the 200–1000 ms time interval after the target stimulus. Behavioral data included the number of target stimuli misses, number of false pressings, reaction time, and reaction time variability. Misses referred to the absence of response in the A-A trials, and false clicks referred to the wrong response in A-P trials. The individual error rates and mean reaction times were averaged over the groups of subjects and compared using the analysis of variance (one-way ANOVA, factor with two levels).

All EEG epochs corresponding to trials with erroneous responses were automatically excluded from further analysis.

### 2.6. Algorithm Description

The problem of schizophrenia diagnostics can be formulated as a binary classification task where classes are the control group and experimental group. In this section, we describe the steps taken for model training.

#### 2.6.1. Feature Engineering

In order to apply machine learning models, one should define features that will be extracted from ERPs. These ERP features will be concatenated with the behavior data and passed through data scaling.

Each ERP signal for a given condition was split into overlapping time windows as suggested in [22]. For each time window, we calculated the minimum, maximum, and average values. Additionally, for each signal, we extracted the global average, global maximum, and global minimum as well as the serial numbers of windows where these extreme values were reached. Since all window features depend on the size of the window and on the shift between windows, the process of feature extraction becomes parametrized. These parameters were selected independently for each paradigm and were treated the same way as the model hyperparameters, and their selection will be described in the last subsection in this section. 

All features will be named using the following template: {condition}_{channel}_{type of feature}_{window number if applicable}, where {type of feature} is one of ‘min’, ‘max’, ‘mean’. For example, feature ‘plus1_T6_min_1′ means the minimal value of the first window of the channel T6 for a condition ‘plus1′.

The resulting number of features can be pretty high depending on the chosen window parameters and can reach up to 1443 features for the considered set of parameters. In order to prevent overfitting, we applied the following techniques for the feature selection:

1. The random normally distributed feature was added to the training dataset. After fitting, the model shap-values [35] were calculated for each feature. All features that had a shap-value less than the random feature were eliminated;
2. Sequential feature selection where the model was trained on the full set of features and after that, the features were eliminated one by one in greedy fashion so that the performance of the model does not decrease;
3. Combination of the above approaches: features with non-random shap-values were selected with successive sequential feature selection;
4. Truncated SVD, which performs the linear dimensionality reduction. The number of features to leave was calculated based on their cumulative explained variance.

Experiments showed that truncated SVD performed worse in our particular case while sequential feature selection could improve the metrics on the hold-out dataset in most cases.

**Figure 4:**
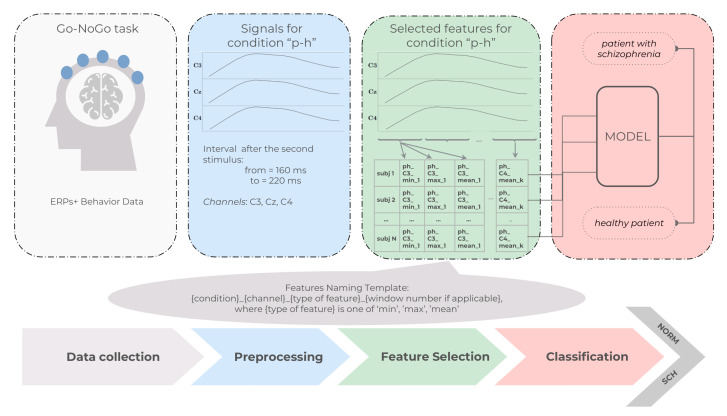
Flowchart showing the stages of data processing for a particular condition ‘P–H’.

Figure 4 depicts a flowchart showing the stages of data processing for a particular condition ‘P–H’. The ERPs and behavior data were collected during the experiment. There is a set of conditions that dictate what signals and what intervals should be taken from the ERPs for further analysis. These chosen signals were passed through the feature selection step, which extracts features according to the parametrized procedure described above. Finally, the extracted features were passed to a classification model.

#### 2.6.2. Considered Models

We considered logistic regression (LR) with L1/L2 regularizations as a baseline model due to its simplicity and ease of interpretation. More complex models that we considered were k-nearest neighbors (kNN), support vector machines (SVM) with various nonlinear kernels, and the stacking model. All models were trained on features described in the previous subsection.

The stacking model uses logistic regressions as the base classifier. Each logistic regression was trained on features from the particular paradigm or on behavior data. The SVM model was trained on probabilities output from logistic regressions as features. Parameters for all of the base models and SVMs were selected through the grid search procedure. The advantage of the stacking model is that we can analyze the feature importance for each paradigm separately. This approach is also more scalable: it allows one to add more feature sources and train models on them without the full retraining of other models. For example, one could consider adding features based on the independent components extracted from the ERPs.

Since our classes were imbalanced (132 individuals in control group vs. 68 individuals in the experimental group), class weighing for all models was used. Aside from class weighing, the use of loss function with class weighing and balancing using upsampling was also implemented for comparison. Obtained results were roughly equivalent so that there was no division between these approaches in the metrics reports.

#### 2.6.3. Pipeline Training

Parameters of the whole pipeline consist of several hyperparameters: parameters of the feature extractor (window size and shift between windows), parameters of the data scaler (what kind of a scaling method to use), and hyperparameters that are specific to a particular model. In order to find the optimal hyperparameters, the grid search technique was applied.

Grid search is a standard tuning technique in machine learning that aims to compute the optimum values of parameters by iterating over the given grid of all possible values of the parameters and training the model on them. The optimal parameters are defined as parameters that lead to better metrics. Grid search was performed on k-fold cross validation in order to obtain a better estimate of the metrics of interest [36]. The number of folds in our case was 10. 

During each grid search step after the features were extracted and scaled according to the current set of parameters, there was an optional step of feature selection before fitting these features to the model. As seen from the experimental results, feature selection improved the model performance in most cases. Sequential feature selection was performed on additional inner cross-validation, that is, the train set of the current fold was additionally split into several folds in order to find the optimal set of features. 

All models were trained in Python using the sklearn package [37].

## 3. Results

### 3.1. Behavioral Parameters

Table 1 shows the behavioral data for each trial type and each experimental group.

The patient group, on average, showed higher miss rates, reaction time, and its variance in comparison to the healthy subjects. An excess of miss rates in patients with schizophrenia had been previously shown [38,39] and it was assumed to indicate a low level of attention to the task and the inability to form a strong prepotent model of response to the target stimuli [38]. Increased reaction time variances in patients with schizophrenia was also described in [40] and might reflect attention lapses [41]. Moreover, in line with [18,19], the present study also found that patients had longer reaction time in Go probes than the healthy controls. Together with the increased miss rate, it might indicate that schizophrenia patients have inefficient cognitive processing.

### 3.2. Model Performance Metrics

In this subsection, the metrics of the resulting models are presented. All metrics were averaged between 10 folds in order to obtain a better estimate.

The main metrics of our interest were sensitivity and specificity. Sensitivity was a true positive rate (TRP), which is a proportion of individuals diagnosed with schizophrenia, who were correctly identified as schizophrenics by the model. Specificity was a true negative rate (TNR) that showed a proportion of healthy people to people who were classified as healthy. Since we needed a single metric in order to be able to compare models with different hyperparameters, the F1 metric was evaluated additionally. Probability thresholds for all models were chosen in such a way that the sensitivity and specificity were roughly the same. Area under the ROC curve (ROC AUC) is another metric that we used that is independent of the probability threshold. The accuracy metric was not evaluated for our models due to class imbalance.

Each model has its own set of features since the number of features is dependent on the parameters selected by grid search for feature extractor and also depends on the features selected by sequential features selection (SFS) and/or features selected after the elimination of features with random shap-value.

In Table 2, the metrics for the models with the best hyperparameters are presented.

The word ‘behavior’ in the parentheses after the name of the model means that the model was trained on a combination of ERP features and behavior data, otherwise, the model was trained on the ERP features only. The abbreviation ‘SFS’ means that feature extraction was followed by sequential features selection, while ‘shap’ means that there was a random feature added to the dataset during the training, and all features with shap-values lower than the shap-value of this random feature were excluded. SFS and shap could be used simultaneously, meaning that sequential feature selection was performed on features with a non-random shap-value. Sequential feature selection can be conducted in a forward fashion where we start training with no features and add features one by one, and in a backward fashion where we start training with a full set of features and eliminate them one by one. Backward SFS is denoted as ‘SFSB’.

Table 2 contains various metric values for the models described in the previous section. For each metric, there were two values in the table: the metric mean value among the cross-validation folds and its standard deviation among folds. The best performance was shown by SVM with no behavioral data and sequential feature selection, and SVM with behavioral data with backward sequential feature selection.

All model hyperparameters were selected through the grid search procedure. The best hyperparameters for each model from Table 2 were as follows: logistic regression had L1 regularization with regularization parameter equal to 1; the kNN model had 11 neighbors; the SVM model had a RBF kernel with the coefficient gamma scaled as 1/(number of features × variance of features) and L2 penalty as a regularization with parameter ⅓.

The model SVM (behavior, SFSB) is described in more detail. The window parameters for various conditions for this model are presented in Table 3.

**Figure 5:**
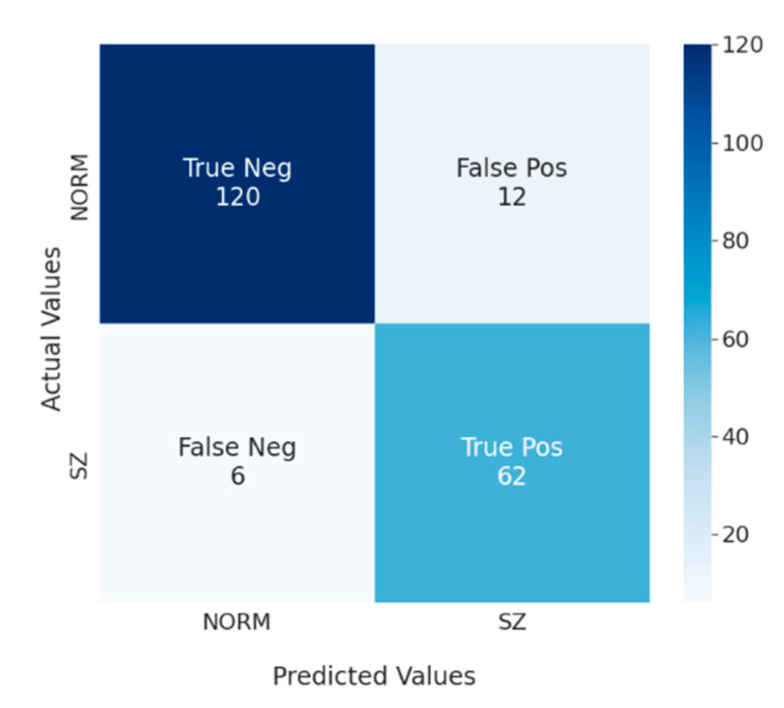
Classification confusion matrix for the model SVM (behavior, SFSB).

Figure 5 represents a confusion matrix for SVM (behavior, SFSB) that was also calculated on the cross-validation. The procedure of its calculation was as follows: on each fold, the model with the best parameters was trained on the training set and counts of true/false negatives/positives were calculated on the test set. Afterward, these counts were summed up. In this way, the resulting confusion matrix takes into account all of data and uses metrics from the test sets only. As can be seen from the matrix, the model correctly identified 121 out of 132 healthy individuals as healthy, classified correctly 62 out of 68 individuals with diagnosed schizophrenia, misclassified 12 healthy people as people with schizophrenia, and six times mistakenly classified individuals diagnosed with schizophrenia as healthy people.

### 3.3. Interpretation

One of the drawbacks of nonlinear models is the complexity of their interpretation. While one can make decisions on the feature importance of linear models by looking at its coefficients for nonlinear models, it requires more effort. In this subsection, the analysis of the resulting models using SHAP (Shapley additive explanations) [35] will be presented. For SHAP calculation, the shap Python library was used [42].

**Figure 6:**
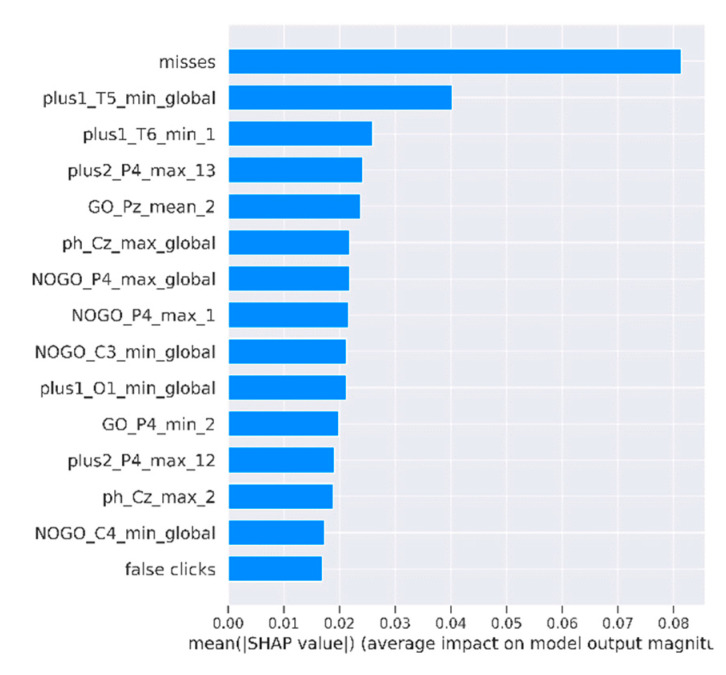
Features sorted based on their shap-value.

The SHAP method allows for the global variance importance to be calculated for each feature. The variance importance of 15 of the most important features of the model SVM (behavior, SFSB) is depicted in Figure 6. Features were sorted by a decrease in their importance on the *Y*-axis. The *X*-axis shows the mean absolute value of SHAP. Names of the ERP features are presented using the template {condition}_{channel}_{type of feature}_{window number if applicable}, where {type of feature} is one of ‘min’, ‘max’, ‘mean’.

**Figure 7:**
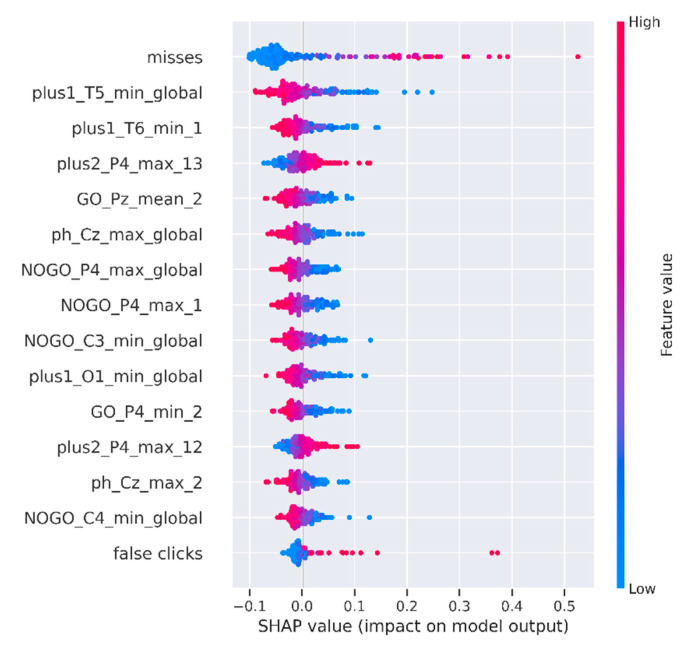
Influence of the feature values on the model output.

Aside from the absolute values of the feature importance, it is interesting to analyze the direction in which each feature has an influence. This visualization is presented in Figure 7. On the *Y*-axis were again the top 15 features sorted by their importance, and on the *X*-axis were the SHAP values. Values to the right from the zero point on the *X*-axis represent a class of schizophrenics, and values to the left are healthy individuals. A red color means a larger value of the feature. The redder the value of the feature, the larger its value. The width of the lines means more observation points. For example, we can see that a small number of misses was more typical for healthy people, while a smaller value of the channel T6 minimum for paradigm ‘plus1′ increased the probability of being diagnosed with schizophrenia.

## 4. Discussion

Most of the studies on the application of machine learning techniques in the diagnosis of schizophrenia used the ERP parameters obtained in the oddball paradigm such as a mismatch negativity, and P300 with accuracy varied between 0.7 and 0.9 [43,44]. Although several studies have explored the late ERP waves in a Go/NoGo paradigm as parameters for discriminating schizophrenic patients from the healthy controls [45], we did not find any papers that had used machine-learning tools to assess the diagnostic power of these parameters.

In the present study, we found that the miss rates had the greatest impact in differentiation between the groups of the healthy control and schizophrenia patients. It was assumed that this indicates a low level of attention to the task and the inability to form a strong prepotent model of response to the target stimuli [38], which are the most obvious symptoms of schizophrenia.

From the ERP parameters, the amplitude of the P3cue wave measured at the T5 and T6 electrodes had a large value in the classification efficacy. This component has been associated with the re-activation of stimulus–response links [46,47].

Many papers have described a decrease in the CNV wave in schizophrenia patients [48,49]. These findings support the claim that CNV amplitude can be used as a criterion for detecting a motivation in patients with schizophrenia.

The decrease in P300 waves in schizophrenia has been reported in numerous studies [18,19,20]. Moreover, the P3b wave, given its association with the allocation of attentional resources to relevant stimuli, is considered as an optimal electrophysiological candidate biomarker of neurocognitive impairment in schizophrenia [50]. The decrease in the P3b amplitude indicate that the effortful allocation of attention to task-relevant stimuli, an important component of decision-making, is compromised in patients with schizophrenia. P300 NOGO modulation is generally considered to reflect an inhibitory process of the prepared action [34], so its decrease in schizophrenia patients is associated with impaired inhibitory control [18,19]. Notably, P300 NOGO over the parietal area (electrode P4) had the greatest impact on classification efficacy between the patients and healthy control, prompting that it was mainly distributed over the frontal-central region.

The P3a wave is usually observed within 200–400 ms after the presentation of infrequent or novel stimuli over the frontal-central cortex. It has been proposed to reflect the redirection of attention toward the rare, distracting stimuli [51] as well as broader alerting. Since this wave is elicited by the presence of new information, it also might represent an updating of the environmental context in light of this new information [52]. P3a deficits in schizophrenia have been reported in several previous papers [52,53]. Furthermore, some studies have demonstrated that the P3a amplitude is reduced in patients with longer illness duration [21]. Therefore, these data suggest that the P3a wave may represent a marker of disease progression, although this suggestion requires further testing [53].

The decrease in the NOGO N2 wave as an index of conflict detection decline in schizophrenia was observed in several studies [45]. However, in the present study, we did not replicate this result. In our previous study, it required the use of a blind source separation method to demonstrate the N2 NOGO reduction in the same group of schizophrenic patients [21].

## 5. Conclusions

We described a novel approach for the classification of schizophrenia from healthy controls using machine learning. Features were extracted from the ERPs of patients performing the visual cued Go/NoGo test. Feature extraction was parametrized and these parameters were selected using the grid search procedure along with the model hyperparameters, followed by the selection of significant features using sequential features selection procedure.

The best model led to values of 91% and 90.8% of sensitivity and specificity, respectively.

The number of misses of the target stimuli, amplitude of the P3cue, and expectancy waves had the greatest impact in group differentiation, which is consistent with the assumption that sustained attention deficit is one of the most significant symptoms of schizophrenia. Surprisingly, the ERP waves with the greatest impact had parietal and temporo-occipital topography, despite the fact that schizophrenia is associated primarily with dysfunction of the frontal cortex. We can hypothesize that in the present study, we observed the result of top–down regulation deficit due to reduced frontal lobe function.

A relatively high percentage of correct classifications allowed us to consider the applied methodology as promising for the development of a tool for diagnosing schizophrenia.

## 6. Limitations and Future Directions

1. Some of the patients were under medication at the time of the EEG recording. Unfortunately, the interruption of medication in patients with severe symptoms could not be implemented due to ethical issues.
2. Another limitation was that the sources of the brain signals were located far from the scalp surface and the respective EEG sensors. Together with volume conductance, it led to overlapping of the brain signals in the EEG recordings. Therefore, we were not able to identify the specific localization of the observed effects based only on the topographies of the EEG potentials. In future studies, we intend to apply the method of extracting latent components of the ERPs [54] to isolate signals from individual brain sources. As shown in the previous papers of the authors [20], the analysis of latent components has the advantage of revealing differences in the parameters of brain responses between groups in intergroup comparisons.

## Figures and Tables

**Table 1:** Behavioral data statistics.

Subject Group	Mean Age	Misses in % Mean ± SE	False Clicks in % Mean ± SE	Reaction Time in % Mean ± SE	Reaction Time Variance Mean ± SE
Healthy	31.8	1.6 ± 2.8	0.7 ± 1.3	379 ± 79	8.4 ± 2.6
Schizophrenia	30.6	9.4 ± 11.4	2.0 ± 6.7	416 ± 92	12.0 ± 4.8
Difference F [1, 198], *p*<	1, 0.32	56.7, 0.001	ns	8.9, 0.01	48.5, 0.001

**Table 2:** Classification performance metrics.

Model	Sensitivity	Specificity	F1	AUC
LR	0.848 ± 0.166	0.855 ± 0.159	0.806 ± 0.159	0.851 ± 0.125
LR (behavior)	0.862 ± 0.132	0.863 ± 0.149	0.820 ± 0.127	0.862 ± 0.097
LR (behavior, SFS)	0.890 ± 0.128	0.885 ± 0.099	0.846 ± 0.079	0.888 ± 0.060
kNN	0.833 ± 0.186	0.856 ± 0.093	0.783 ± 0.134	0.845 ± 0.101
kNN (behavior)	0.867 ± 0.175	0.871 ± 0.098	0.815 ± 0.137	0.869 ± 0.102
kNN (behavior, SFS)	0.895 ± 0.130	0.916 ± 0.054	0.867 ± 0.098	0.906 ± 0.075
Stacking (behavior)	0.862 ± 0.116	0.871 ± 0.118	0.822 ± 0.124	0.866 ± 0.093
SVM	0.893 ± 0.121	0.886 ± 0.103	0.849 ± 0.125	0.890 ± 0.090
SVM (SFS)	0.912 ± 0.096	0.908 ± 0.083	0.877 ± 0.098	0.910 ± 0.074
SVM (shap, SFS)	0.895 ± 0.094	0.901 ± 0.103	0.863 ± 0.079	0.898 ± 0.055
SVM (SFSB)	0.907 ± 0.124	0.910 ± 0.091	0.872 ± 0.108	0.909 ± 0.080
SVM (behavior)	0.907 ± 0.107	0.902 ± 0.082	0.866 ± 0.091	0.904 ± 0.067
SVM (behavior, SFS)	0.895 ± 0.114	0.896 ± 0.097	0.854 ± 0.107	0.895 ± 0.080
SVM (behavior, shap, SFS)	0.876 ± 0.138	0.878 ± 0.116	0.833 ± 0.095	0.877 ± 0.072
SVM (behavior, SFSB)	0.910 ± 0.074	0.908 ± 0.083	0.877 ± 0.091	0.909 ± 0.067

**Table 3:** Window parameters of the model SVM (behavior, SFSB) with the best hyperparameters.

Condition	Window Size in ms	Window Shift in %
plus 1	49	50
plus 2	5	50
NO-GO	49	100
GO	20	100
P-H	5	100

## Data Availability

Data sharing not applicable.

## References

[1] Owen M., Sawa A., Mortensen P. (2016). Schizophrenia. Lancet.

[2] McEvoy J.P. (2007). The importance of early treatment of schizophrenia. Behav. Health.

[3] Bowie C.R., Harvey P.D. (2006). Cognitive deficits and functional outcome in schizophrenia. Neuropsychiatr. Dis. Treat..

[4] Orellana G., Slachevsky A., Peña M. (2012). Executive attention impairment in first-episode schizophrenia. BMC Psychiatry.

[5] Rinaldi R., Lefebvre L. (2016). Goal-directed behaviors in patients with schizophrenia: Concept relevance and updated model. Psychiatry Clin. Neurosci..

[6] Mayer A.R., Hanlon F.M., Dodd A.B., Yeo R.A., Haaland K.Y., Ling J.M., Ryman S.G. (2016). Proactive response inhibition abnormalities in the sensorimotor cortex of patients with schizophrenia. J. Psychiatry Neurosci..

[7] Perez V.B., Ford J.M., Roach B.J., Woods S.W., McGlashan T.H., Srihari V.H., Loewy R.L., Vinogradov S., Mathalon D.H. (2012). Error monitoring dysfunction across the illness course of schizophrenia. J. Abnorm. Psychol..

[8] Cadena E.J., White D.M., Kraguljac N.V., Reid M.A., Lahti A.C. (2018). Evaluation of fronto-striatal networks during cognitive control in unmedicated patients with schizophrenia and the effect of antipsychotic medication. Schizophrenia.

[9] Battaglia S., Cardellicchio P., Di Fazio C., Nazzi C., Fracasso A., Borgomaneri S. (2022). Stopping in (e)motion: Reactive action inhibition when facing valence-independent emotional stimuli. Front. Behav. Neurosci..

[10] Warren S.L., Crocker L.D., Spielberg J.M., Engels A.S., Banich M.T., Sutton B.P., Miller G.A., Heller W. (2013). Cortical organization of inhibition-related functions and modulation by psychopathology. Front. Hum. Neurosci..

[11] Schröder J., Wenz F., Schad L.R., Baudendistel K., Knopp M.V. (1995). Sensorimotor Cortex and Supplementary Motor Area Changes in Schizophrenia: A Study with Functional Magnetic Resonance Imaging. Br. J. Psychiatry.

[12] Akar S.A., Kara S., Latifoğlu F., Bilgiç V. (2016). Analysis of the Complexity Measures in the EEG of Schizophrenia Patients. Int. J. Neural Syst..

[13] Kutepov I.E., Krysko V.A., Krysko A.V., Pavlov S.P., Zigalov M.V., Papkova I.V., Saltykova O.A., Yaroshenko T.Y., Krylova E.Y., Yakovleva T.V. Complexity of EEG Signals in Schizophrenia Syndromes. Proceedings of the 29th International Conference on Computer Graphics and Vision.

[14] Zhang L. EEG Signals Classification Using Machine Learning for The Identification and Diagnosis of Schizophrenia. Proceedings of the 41st Annual International Conference of the IEEE Engineering in Medicine and Biology Society (EMBC).

[15] Kropotov J.D., Mueller A. (2009). What can event related potentials contribute to neuropsychology. Acta Neuropsychol..

[16] Kropotov J.D., Pronina M.V., Ponomarev V.A., Murashev P.V. (2011). In Search of New Protocols of Neurofeedback: Independent Components of Event-Related Potentials. J. Neurother..

[17] DeLaRosa B.L., Spence J.S., Motes M.A., To W., Vanneste S., Kraut M.A., Hart J. (2020). Identification of selection and inhibition components in a Go/NoGo task from EEG spectra using a machine learning classifier. Brain Behav..

[18] Ertekin E., Üçok A., Keskin-Ergen Y., Devrim-Üçok M. (2017). Deficits in Go and NoGo P3 potentials in patients with schizophrenia. Psychiatry Res..

[19] Sun Q., Fang Y., Shi Y., Wang L., Peng X., Tan L. (2021). Inhibitory Top-Down Control Deficits in Schizophrenia With Auditory Verbal Hallucinations: A Go/NoGo Task. Front. Psychiatry.

[20] Kropotov J.D., Pronina M.V., Ponomarev V.A., Poliakov Y.I., Plotnikova I.V., Mueller A. (2019). Latent ERP components of cognitive dysfunctions in ADHD and schizophrenia. Clin. Neurophysiol..

[21] Oribe N., Hirano Y., Kanba S., Del Re E., Seidman L., Mesholam-Gately R., Goldstein J.M., Shenton M., Spencer K.M., McCarley R.W. (2015). Progressive reduction of visual P300 amplitude in patients with first-episode schizophrenia: An ERP study. Schizophr. Bull..

[22] Mueller A., Candrian G., Kropotov J.D., Ponomarev V.A., Baschera G.-M. (2010). Classification of ADHD patients on the basis of independent ERP components using a machine learning system. Nonlinear Biomed. Phys..

[23] Müller A., Vetsch S., Pershin I., Candrian G., Baschera G.-M., Kropotov J.D., Kasper J., Rehim H.A., Eich D. (2019). EEG/ERP-based biomarker/neuroalgorithms in adults with ADHD: Development, reliability, and application in clinical practice. World J. Biol. Psychiatry.

[24] Franke G.H. (2000). BSI. Brief Symptom Inventory—Deutsche Version. Manual.

[25] Barkley R.A., Murphy K.R. (2006). Attention-Deficit Hyperactivity Disorder: A Clinical Workbook.

[26] Kay S.R., Fiszbein A., Opler L.A. (1987). The Positive and Negative Syndrome Scale (PANSS) for Schizophrenia. Schizophr. Bull..

[27] Jung T.-P., Makeig S., Westerfield M., Townsend J., Courchesne E., Sejnowski T.J. (2000). Removal of eye activity artifacts from visual event-related potentials in normal and clinical subjects. Clin. Neurophysiol..

[28] Ponomarev V.A., Mueller A., Candrian G., Grin-Yatsenko V.A., Kropotov J.D. (2014). Group Independent Component Analysis (gICA) and Current Source Density (CSD) in the study of EEG in ADHD adults. Clin. Neurophysiol..

[29] Karayanidis F., Jamadar S., Ruge H., Phillips N., Heathcote A., Forstmann B.U. (2010). Advance preparation in task-switching: Converging evidence from behavioral, brain activation, and model-based approaches. Front. Psychol..

[30] De Baene W., Albers A.M., Brass M. (2012). The what and how components of cognitive control. Neuroimage.

[31] Aydin M., Carpenelli A.L., Lucia S., Di Russo F. (2022). The Dominance of Anticipatory Prefrontal Activity in Uncued Sensory–Motor Tasks. Sensors.

[32] Polich J. (2007). Updating P300: An integrative theory of P3a and P3b. Clin. Neurophysiol..

[33] Huang W.-J., Chen W.-W., Zhang X. (2015). The neurophysiology of P 300—An integrated review. Eur. Rev. Med. Pharmacol. Sci..

[34] Bokura H., Yamaguchi S., Kobayashi S. (2001). Electrophysiological correlates for response inhibition in a Go/NoGo task. Clin. Neurophysiol..

[35] Štrumbelj E., Kononenko I. (2013). Explaining prediction models and individual predictions with feature contributions. Knowl. Inf. Syst..

[36] Borra S., Di Ciaccio A. (2010). Measuring the prediction error. A comparison of cross-validation, bootstrap and covariance penalty methods. Comput. Stat. Data Anal..

[37] Pedregosa F., Varoquaux G., Gramfort A., Michel V., Thirion B., Grisel O., Blondel M., Prettenhofer P., Weiss R., Dubourg V. (2011). Scikit-learn: Machine Learning in Python. JMLR.

[38] Ford J.M., Mathalon D.H. (2003). Electrophysiological evidence of corollary discharge dysfunction in schizophrenia during talking and thinking. J. Psychiatr. Res..

[39] Pallanti S., Salerno L. (2015). Raising attention to attention deficit hyperactivity disorder in schizophrenia. World J. Psychiatry.

[40] Kaiser S., Roth A., Rentrop M., Friederich H.C., Bender S., Weisbrod M. (2008). Intra-individual reaction time variability in schizophrenia, depression and borderline personality disorder. Brain Cogn..

[41] Weissman D.H., Roberts K.C., Visscher K.M., Woldorff M. (2006). The neural bases of momentary lapses in attention. Nat. Neurosci..

[42] Lundberg S., Lee S.-I. A Unified Approach to Interpreting Model Predictions. Proceedings of the 31st International Conference on Neural Information Processing Systems—NIPS’17.

[43] Barros C., Silva C.A., Pinheiro A.P. (2021). Advanced EEG-based learning approaches to predict schizophrenia: Promises and pitfalls. Artif. Intell. Med..

[44] Santos F.E., Ontivero O.M., Valdés S.M., Sahli H. (2022). Machine Learning Techniques for the Diagnosis of Schizophrenia Based on Event-Related Potentials. Front. Neuroinform..

[45] Hoonakker M., Doignon-Camus N., Marques-Carneiro J.E., Bonnefond A. (2017). Sustained attention ability in schizophrenia: Investigation of conflict monitoring mechanisms. Clin. Neurophysiol..

[46] Barceló F., Periáñez J.A., Nyhus E. (2008). An information theoretical approach to task-switching: Evidence from cognitive brain potentials in humans. Front. Hum. Neurosci..

[47] Gajewski P.D., Falkenstein M. (2011). Diversity of the P3 in the task-switching paradigm. Brain Res..

[48] Lin Y.X., Zhang L.J., Ying L., Zhou Q. (2020). Cognitive effort-avoidance in patients with schizophrenia can reflect Amotivation: An event-related potential study. BMC Psychiatry.

[49] Catalano L.T., Wynn J.K., Lee J., Green M.F. (2021). A comparison of stages of attention for social and nonsocial stimuli in schizophrenia: An ERP study. Schizophr. Res..

[50] Giordano G.M., Perrottelli A., Mucci A., Di Lorenzo G., Altamura M., Bellomo A., Brugnoli R., Corrivetti G., Girardi P., Monteleone P. (2021). Investigating the Relationships of P3b with Negative Symptoms and Neurocognition in Subjects with Chronic Schizophrenia. Brain Sci..

[51] Escera C., Alho K., Winkler I., Näätänen R. (1998). Neural Mechanisms of Involuntary Attention to Acoustic Novelty and Change. J. Cogn. Neurosci..

[52] Fisher D.J., Campbell D.J., Abriel S.C., Ells E.M.L., Rudolph E.D., Tibbo P.G. (2018). Auditory Mismatch Negativity and P300a Elicited by the “Optimal” Multi-feature Paradigm in Early Schizophrenia. Clin. EEG Neurosci..

[53] Giordano G.M., Giuliani L., Perrottelli A., Bucci P., Di Lorenzo G., Siracusano A., Brando F., Pezzella P., Fabrazzo M., Altamura M. (2021). Mismatch Negativity and P3a Impairment through Different Phases of Schizophrenia and Their Association with Real-Life Functioning. J. Clin. Med..

[54] Metsomaa J., Sarvas J., Ilmoniemi R.J. (2016). Blind Source Separation of Event-Related EEG/MEG. IEEE Trans. Biomed. Eng..

